# The Epidemiology of US Upper Extremity Amputations: An Analysis of the Global Burden of Disease Database (1990-2019)

**DOI:** 10.7759/cureus.97244

**Published:** 2025-11-19

**Authors:** Ambrose Loc T Ngo, Cameron J Sabet, Gabrielle Dykhouse, Ayah Ibrahim, Taylor J Manes, Arsalaan Sayyed, Shaheryar Asad, Benjamin C Taylor

**Affiliations:** 1 College of Osteopathic Medicine, Kansas City University, Joplin, USA; 2 Department of Surgery, Georgetown University School of Medicine, Washington, DC, USA; 3 School of Medicine, Cornell University, Ithaca, USA; 4 Department of Orthopedics, Burrell College of Osteopathic Medicine, Las Cruces, USA; 5 Department of Orthopedic Surgery, OhioHealth Doctors Hospital, Columbus, USA; 6 School of Osteopathic Medicine, Campbell University School of Osteopathic Medicine, Lillington, USA; 7 College of Medicine, California Northstate University College of Medicine, Elk Grove, USA; 8 Department of Orthopedic Trauma, OhioHealth Grant Medical Center, Columbus, USA

**Keywords:** ambulatory surgery, health public, major limb amputation, ortho surgery, surgery general

## Abstract

Background

Upper extremity amputations (UEAs) are medically, psychologically, and functionally devastating. This study aimed to evaluate region- and sex-specific differences in unilateral and bilateral upper extremity amputations in the United States (US) from 1990 to 2019.

Methods

The Global Burden of Disease (GBD) database was used to analyze years lived with disability (YLDs), prevalence, and incidence rates per 100,000 people for upper extremity amputations in the US from 1990 to 2019. Data were stratified into four US Census Bureau-defined regions: Northeast, Midwest, South, and West. Differences between regions and sexes were assessed, with statistical significance defined as p<0.05.

Results

Between 1990 and 2019, unilateral upper extremity amputations in the US showed a 34.51% decrease in YLDs, 34.34% in prevalence, and 37.38% in incidence. Bilateral amputations decreased by 51.32%, 48.94%, and 56.80%, respectively. Men consistently had higher YLDs, prevalence, and incidence rates for unilateral amputations (p<0.001). Regionally, the Northeast had the highest mean YLDs, prevalence, and incidence, while the South had the lowest. Men also experienced significantly higher rates of both unilateral and bilateral amputations across all regions compared to women (p<0.05).

Conclusions

From 1990 to 2019, the US experienced declines in YLDs, prevalence, and incidence of upper extremity amputations. Men had higher rates than women across all regions. The Northeast exhibited the highest rates, while the South had the lowest. These findings underscore notable gender and regional disparities in upper extremity amputation trends.

## Introduction

Upper extremity amputations (UEAs) bring serious disruption to the functional and psychological well-being of 41,000 new patients annually in the United States (US) [1,2]. Overall, it is estimated that 1.4 million people in the US have limb loss [2]. Researchers have investigated the prevalence and relative burden of limb amputations across various demographics in multiple contexts. For instance, one research team studied hand and wrist trauma using the Global Burden of Disease (GBD) database, including the prevalence of fractures and digit amputations worldwide [3]. Their findings revealed considerable regional variation and several relevant etiological factors, including falls, machinery-related injuries, and automobile accidents [4]. In general, upper extremity amputations arise from a range of causes, with trauma being the predominant factor in younger and working-age adults, while vascular complications, infections, and malignant tumors contribute more substantially among older or medically complex populations [4,5]. Researchers have also studied major traumatic limb amputations and their outcomes to advocate for appropriate rehabilitation and prosthetic services for all populations, including the military [1,5,6]. Despite these contributions to amputation science, there is a clear absence of epidemiological research into the general population of all UEAs in the US specifically. Although global studies provide a relevant view from the universal distribution of amputations, they miss important nuances that uniquely impact the upper extremities in the US. These differences have a diversity and a temporality specific to Americans. Neither of these nuances has been examined at a population level over a sufficiently long period of time.

This study aims to perform a temporal epidemiological analysis of UEAs in the US from 1990 to 2019, using the data available from the GBD database. This aim will be achieved by analyzing the trends in incidence, prevalence, and related factors of UEAs from 1990 to 2019 to try to explain the evolution of these procedures and inform healthcare systems of guidelines to improve future outcomes for the individuals affected. In addition, we will identify regional differences and explore the etiology of these amputations.

## Materials and methods

Study design and overview

This was a retrospective, population-based epidemiological study analyzing upper extremity amputation (UEA) trends across the United States from 1990 to 2019. All data were obtained from publicly accessible datasets; therefore, institutional review board (IRB) approval was not required. The study followed the Strengthening the Reporting of Observational Studies in Epidemiology (STROBE) guidelines for cross-sectional analyses.

Data sources

Epidemiological data were extracted from the Global Burden of Disease (GBD) dataset developed by the Institute for Health Metrics and Evaluation (IHME) [7,8]. The GBD dataset provides comprehensive estimates of the incidence, prevalence, mortality, and disability burden associated with 369 diseases and injuries across 204 countries between 1990 and 2019. The dataset integrates multiple data sources, including administrative health records, hospital discharge databases, national census data, population-based surveys, and geospatial and demographic modeling data. The GBD employs standardized statistical modeling techniques to adjust for underreporting and sampling bias. Its methods and validation protocols have been described in prior publications [9-11]. For the present study, US data were stratified into four geographic regions, Northeast, Midwest, South, and West, as defined by the US Census Bureau [12,13].

Study variables and outcomes

The primary outcomes included years lived with disability (YLD), incidence (new cases per year), and prevalence (total existing cases). All rates were age-standardized per 100,000 persons and analyzed separately for men and women across each US state and by region. YLDs were defined according to the World Health Organization as “one full year of healthy life lost due to disability or ill-health” [14]. These measures reflect the non-fatal burden of disease and provide insight into chronic disability patterns related to upper extremity amputations.

Data processing

Data were downloaded in tabular format from the IHME GBD Results Tool. Records were screened for completeness and aggregated by sex, region, and year. Where applicable, missing or anomalous values were cross-referenced with previous GBD iterations or interpolated using IHME’s published data hierarchy protocols to ensure consistency across the study period.

Statistical analysis

All statistical analyses were performed using IBM SPSS Statistics version 29 (SPSS Inc., Chicago, IL). The analytical framework followed previously published and validated methods [15]. Bartlett’s test was first conducted to evaluate the homogeneity of variance across all datasets. For measures demonstrating unequal variance, Welch’s analysis of variance (ANOVA) was used to assess regional differences in years lived with disability (YLDs), prevalence, and incidence, followed by the Games-Howell post hoc test for multiple comparisons. For datasets with equal variance, a standard ANOVA was performed with Tukey’s post hoc analysis. Additionally, independent t-tests were used to compare mean YLDs, prevalence, and incidence rates between men and women at both the regional and national levels. All results were considered statistically significant at a two-tailed p-value of <0.05.

Ethical considerations

This study utilized publicly available, de-identified data; therefore, ethical approval and informed consent were not required.

## Results

United States and data by sex

**Figure 1:**
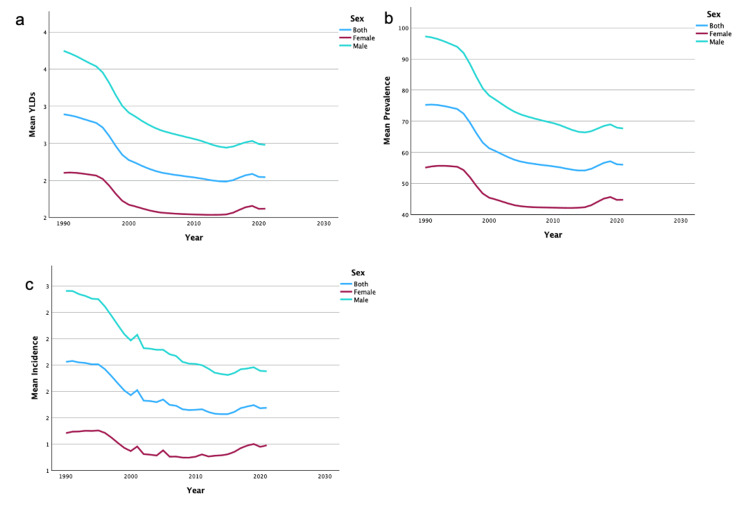
Temporal trends of YLDs, prevalence, and incidence for unilateral upper extremity amputations by sex These figures illustrate temporal trends from 1990 to 2019 in mean YLDs (1a), mean prevalence (1b), and mean incidence (1c) of unilateral upper extremity amputations, stratified by sex. YLDs: years lived with disability

**Figure 2:**
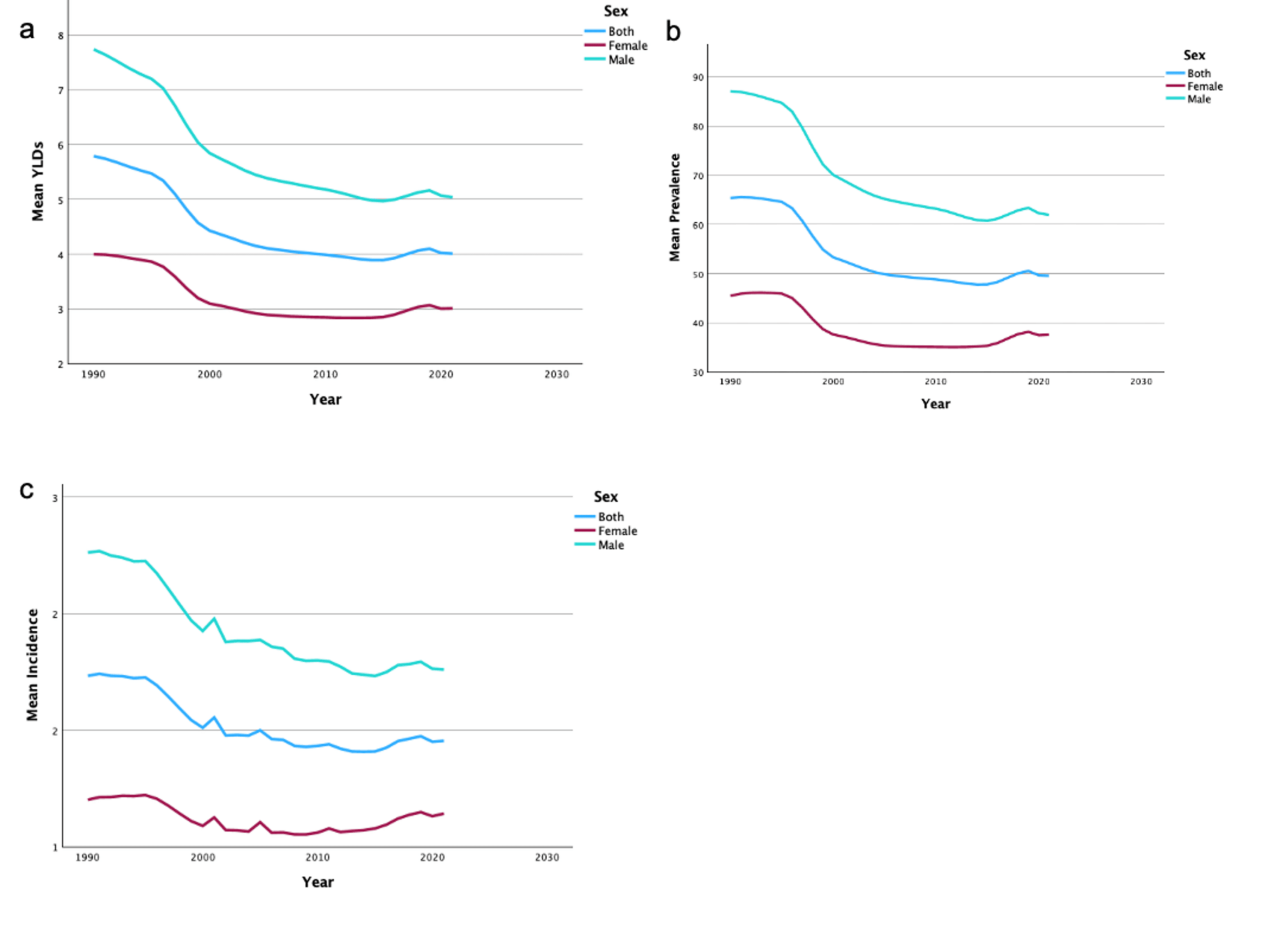
Temporal trends of YLDs, prevalence, and incidence for bilateral upper extremity amputations by sex These figures illustrate temporal trends from 1990 to 2019 in mean YLDs (2a), mean prevalence (2b), and mean incidence (2c) of bilateral upper extremity amputations, stratified by sex. YLDs: years lived with disability

From 1990 to 2019, the US saw a 34.51% decrease in YLDs, a 34.34% decrease in the prevalence rate, and a 37.38% decrease in the incidence rate of unilateral UEAs. In addition, the US saw a 51.32% decrease in YLDs, 48.94% in prevalence rate, and a 56.80% decrease in incidence rate for bilateral amputations. Regardless of region or sex, there was a decrease in overall mean YLDs, incidence, and prevalence of UEAs from 1990 to 2019. A summary of the temporal trends of YLD, incidence, and prevalence for both sexes for unilateral UEA can be found in Figures 1a-1c. A summary of the temporal trends of YLD, incidence, and prevalence for both sexes for bilateral UEA can be found in Figures 2a-2c. Women never surpassed men in YLDs, incidence, and prevalence of unilateral and bilateral UEA between 1990 and 2019.

Data by region

**Figure 3:**
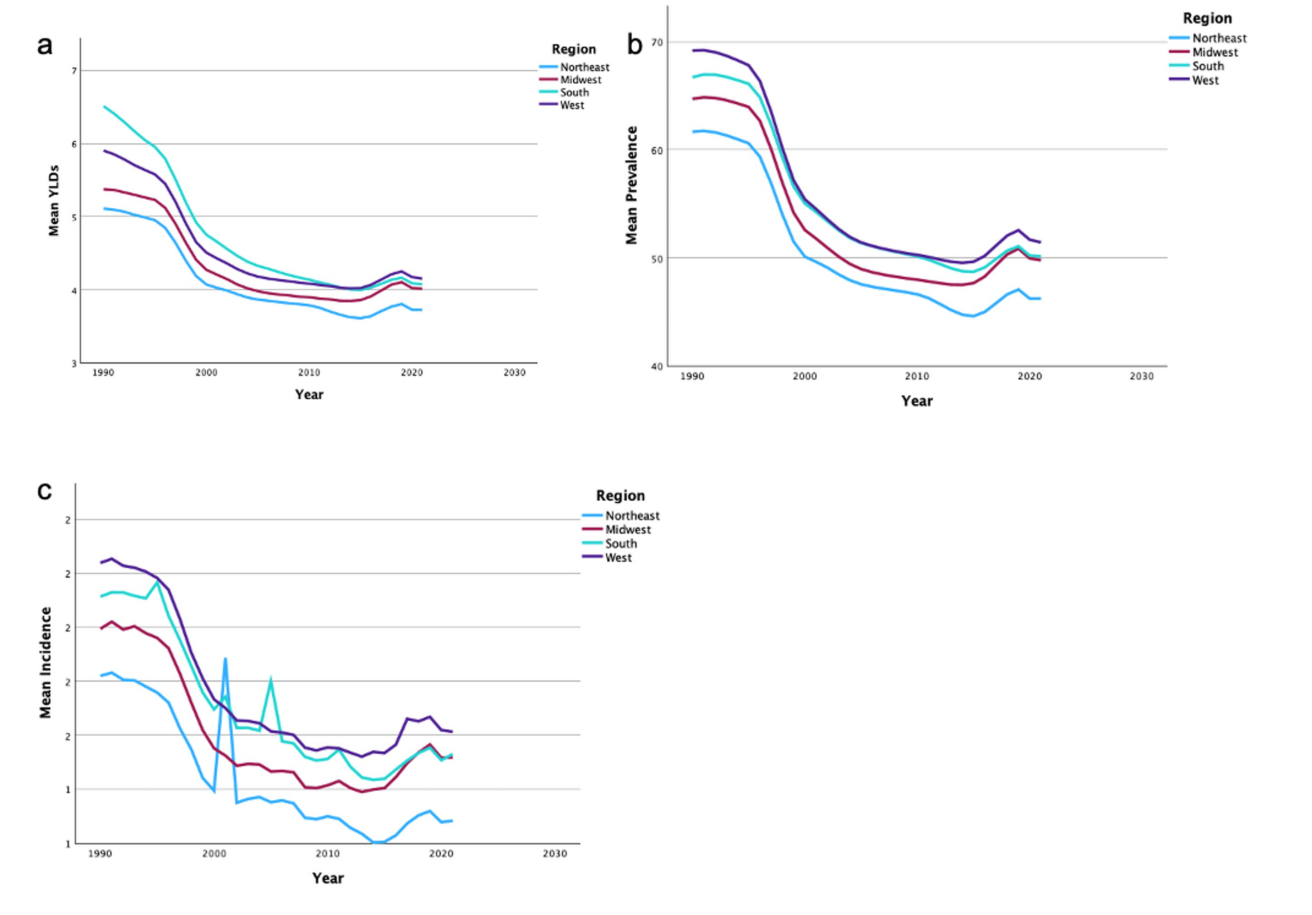
Temporal trends of YLDs, prevalence, and incidence for bilateral upper extremity amputations by region The figures present the temporal trends from 1990 to 2019 in mean YLDs (3a), mean prevalence (3b), and mean incidence (3c) of bilateral upper extremity amputations, categorized by regions: Northeast, Midwest, South, and West. YLDs: years lived with disability

**Figure 4:**
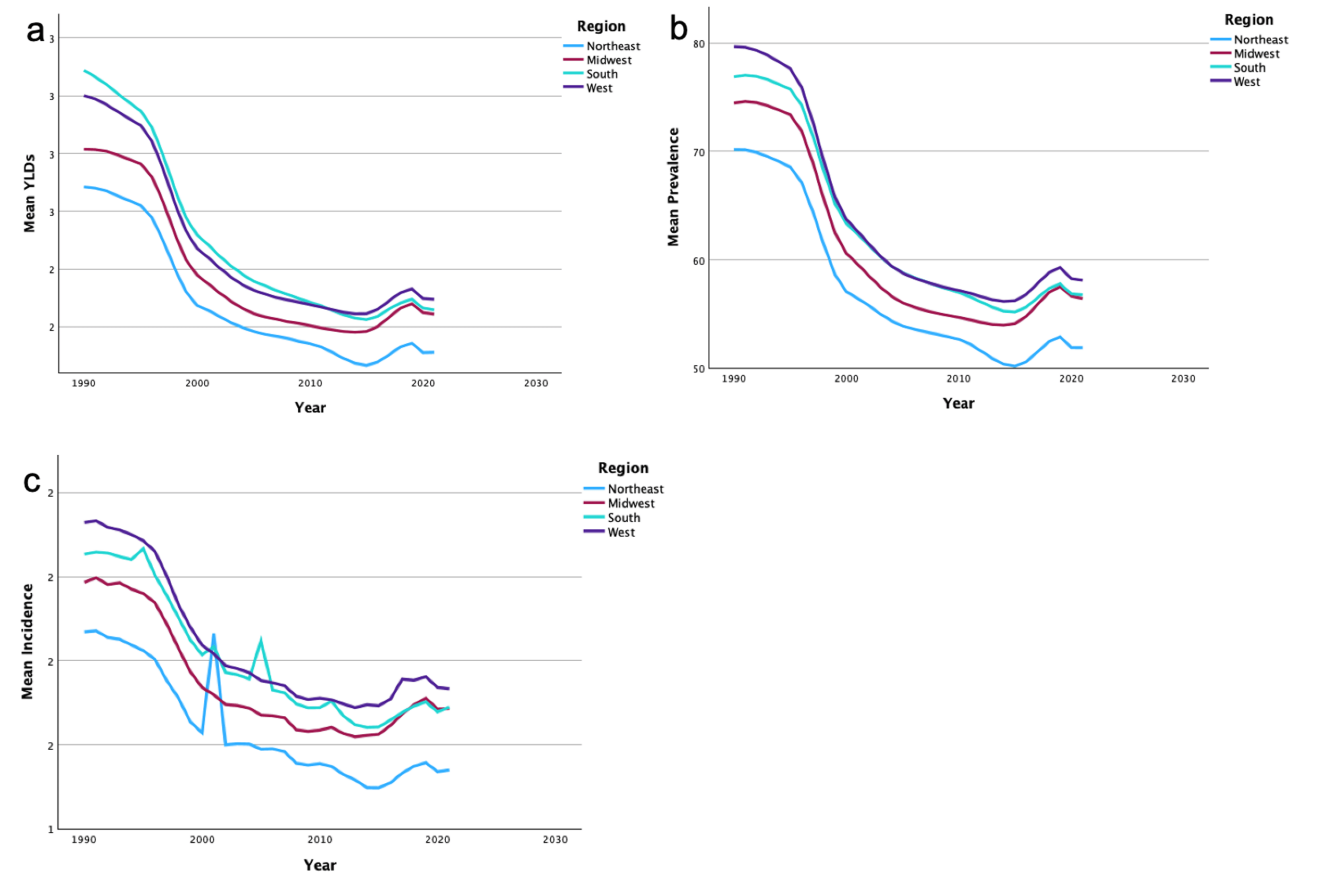
Temporal trends of YLDs, prevalence, and incidence for unilateral upper extremity amputations by region The figures present the temporal trends from 1990 to 2019 in mean YLDs (4a), mean prevalence (4b), and mean incidence (4c) of unilateral upper extremity amputations, categorized by regions: Northeast, Midwest, South, and West. YLDs: years lived with disability

A summary of the temporal trends of YLD, incidence, and prevalence for bilateral UEA in each region can be found in Figures 3a-3c. Additionally, a summary of the temporal trends of YLD, incidence, and prevalence for unilateral UEA in each region can be found in Figures 4a-4c. Regional analysis demonstrated that the Northeast region had the highest overall mean YLDs in bilateral UEA. The West region had the highest overall mean YLDs in unilateral UEA. The Northeast region also demonstrated the highest overall mean incidence and prevalence of bilateral and unilateral UEA. The South region had the lowest overall mean in YLDs, incidence, and prevalence of unilateral UEA. The South region also had the lowest overall mean in YLD in bilateral UEA. Finally, the Midwest region had the lowest overall mean in incidence and prevalence of bilateral UEA.

From 1990 to 2019, the Northeast region saw a 36.09% decrease in YLDs, a 35.36% decrease in the prevalence rate, and a 39.49% decrease in the incidence rate of unilateral UEAs. In addition, the Northeast region also saw a 54.50% decrease in YLDs, a 50.84% decrease in prevalence rate, and a 58.22% decrease in incidence rate for bilateral amputations. The Midwest region saw a 33.72% decrease in YLDs, a 34.64% decrease in the prevalence rate, and a 36.79% decrease in the incidence rate of unilateral UEAs. In addition, the Midwest region also saw a 48.34% decrease in YLDs, a 46.19% decrease in prevalence rate, and a 54.89% decrease in incidence rate for bilateral amputations. The South region saw a 31.69% decrease in YLDs, a 31.46% decrease in the prevalence rate, and a 34.16% decrease in the incidence rate of unilateral UEAs. In addition, the South region also saw a 48.19% decrease in YLDs, a 48.19% decrease in prevalence rate, and a 55.79% decrease in incidence rate for bilateral amputations. Finally, the West region saw a 37.45% decrease in YLDs, a 35.76% decrease in the prevalence rate, and a 38.84% decrease in the incidence rate of unilateral UEAs. In addition, the West region also saw a 53.74% decrease in YLDs, a 50.21% decrease in prevalence rate, and a 56.92% decrease in incidence rate for bilateral amputations. Men were more likely to experience higher YLDs, incidence, and prevalence of both unilateral and bilateral UEA in each of the regions compared to women (p<0.05).

Data by state

A summary of percent change from 1990 to 2019 for all outcome measures in all states can be found in Table 1 for bilateral UEA and Table 2 for unilateral UEA. The District of Columbia experienced the most significant decrease in the rates of YLDs for bilateral UEA (61.60%) from 1990 to 2019. North Dakota experienced the least significant decrease in YLDs for bilateral UEA (16.17%). California displayed the most significant decrease in YLDs, prevalence, and incidence rate for unilateral UEAs. Illinois experienced the smallest decrease in YLDs, prevalence, and incidence for unilateral UEAs.

**Table 1:** Percent change from 1990 to 2019 of YLDs, prevalence, and incidence for bilateral UEAs YLDs: years lived with disability, UEAs: upper extremity amputations

State	YLDs 1990	YLDs 2021	% change	Prevalence 1990	Prevalence 2021	% change	Incidence 1990	Incidence 2021	% change
Alabama	6.89	4.17	-39.56	68.09	51.49	-24.38	1.80	1.49	-17.48
Alaska	7.37	4.62	-37.33	79.72	57.15	-28.31	2.12	1.66	-21.63
Arizona	5.59	4.19	-25.01	68.63	51.98	-24.27	1.86	1.56	-16.14
Arkansas	6.91	4.56	-34.10	72.25	56.58	-21.69	1.92	1.67	-12.65
California	5.79	3.65	-36.92	63.21	44.86	-29.04	1.67	1.29	-22.51
Colorado	5.67	4.35	-23.32	69.49	54.04	-22.24	1.87	1.64	-12.71
Connecticut	4.94	3.52	-28.64	60.35	43.71	-27.57	1.58	1.27	-19.47
Delaware	5.45	3.68	-32.49	61.52	45.60	-25.87	1.62	1.31	-19.03
District of Columbia	10.51	4.03	-61.60	63.20	47.86	-24.28	1.71	1.48	-13.55
Florida	5.42	3.96	-26.97	66.52	49.15	-26.11	1.77	1.43	-18.80
Georgia	6.97	3.82	-45.16	65.27	47.47	-27.28	1.74	1.39	-20.33
Hawaii	4.94	3.77	-23.65	60.50	46.53	-23.10	1.59	1.34	-15.76
Idaho	5.58	4.04	-27.47	68.19	49.96	-26.74	1.79	1.46	-18.16
Illinois	5.80	3.64	-37.31	65.18	44.93	-31.07	1.73	1.30	-24.84
Indiana	5.11	3.79	-25.84	62.56	47.02	-24.85	1.64	1.35	-17.39
Iowa	5.05	3.99	-20.93	61.86	49.41	-20.13	1.64	1.47	-10.56
Kansas	5.29	4.05	-23.49	64.76	50.31	-22.32	1.71	1.48	-13.66
Kentucky	5.45	4.13	-24.22	67.02	51.46	-23.22	1.79	1.51	-15.48
Louisiana	6.49	4.14	-36.27	66.43	51.58	-22.37	1.76	1.46	-16.72
Maine	5.03	3.88	-22.83	61.51	48.27	-21.52	1.62	1.42	-12.55
Maryland	5.70	4.01	-29.73	63.51	49.39	-22.24	1.67	1.44	-14.14
Massachusetts	5.06	3.88	-23.26	62.05	48.06	-22.55	1.64	1.39	-15.26
Michigan	5.40	3.63	-32.77	59.56	45.22	-24.08	1.56	1.31	-16.11
Minnesota	5.37	4.00	-25.48	65.64	49.41	-24.72	1.75	1.48	-15.37
Mississippi	7.51	4.71	-37.30	68.02	52.79	-22.38	1.81	1.53	-15.15
Missouri	5.66	4.15	-26.73	68.89	51.52	-25.21	1.83	1.55	-15.36
Montana	5.81	4.63	-20.37	71.34	57.37	-19.58	1.85	1.65	-10.67
Nebraska	5.30	4.08	-23.08	64.98	50.56	-22.20	1.72	1.48	-14.10
Nevada	6.54	4.19	-35.87	72.10	52.02	-27.84	1.91	1.48	-22.72
New Hampshire	5.04	3.88	-23.00	61.72	48.07	-22.10	1.63	1.41	-13.64
New Jersey	4.64	3.44	-25.76	56.86	42.61	-25.07	1.49	1.22	-18.41
New Mexico	7.24	4.50	-37.77	74.12	56.14	-24.26	1.99	1.71	-13.90
New York	5.48	3.38	-38.23	59.11	41.95	-29.04	1.56	1.19	-23.41
North Carolina	6.05	3.76	-37.77	63.61	46.73	-26.53	1.70	1.42	-16.78
North Dakota	5.06	4.24	-16.17	61.92	52.64	-14.98	1.62	1.51	-6.91
Ohio	5.06	3.74	-26.17	61.94	46.56	-24.83	1.65	1.38	-16.12
Oklahoma	5.61	4.40	-21.58	68.08	55.00	-19.21	1.87	1.62	-13.36
Oregon	5.62	3.98	-29.11	68.64	49.26	-28.24	1.83	1.47	-19.71
Pennsylvania	4.95	3.70	-25.33	60.82	45.74	-24.80	1.60	1.34	-15.81
Rhode Island	5.01	3.64	-27.19	61.25	45.34	-25.98	1.62	1.34	-17.69
South Carolina	7.06	3.94	-44.17	64.15	48.99	-23.63	1.70	1.42	-16.41
South Dakota	5.54	4.63	-16.32	67.58	57.17	-15.40	1.79	1.66	-7.24
Tennessee	6.26	3.98	-36.49	66.35	49.50	-25.40	1.75	1.47	-15.97
Texas	5.98	3.88	-35.13	69.27	47.93	-30.81	1.85	1.40	-24.15
Utah	5.20	3.86	-25.73	63.57	47.98	-24.52	1.68	1.42	-15.74
Vermont	5.39	4.09	-24.04	65.55	50.71	-22.64	1.75	1.52	-13.37
Virginia	5.12	3.74	-26.93	62.34	46.24	-25.82	1.67	1.35	-18.99
Washington	5.35	3.75	-29.89	65.62	46.36	-29.35	1.74	1.39	-20.24
West Virginia	5.84	4.01	-31.27	63.85	50.14	-21.46	1.68	1.49	-11.13
Wisconsin	5.30	4.17	-21.27	64.84	51.78	-20.14	1.73	1.57	-9.28
Wyoming	5.83	4.47	-23.26	71.00	55.21	-22.23	1.85	1.59	-14.11

**Table 2:** Percent change from 1990 to 2019 of YLDs, prevalence, and incidence for unilateral UEAs YLDs: years lived with disability, UEAs: upper extremity amputations

State	YLDs 1990	YLDs 2021	% change	Prevalence 1990	Prevalence 2021	% change	Incidence 1990	Incidence 2021	% change
Alabama	3.27	2.15	-34.30	79.24	59.03	-25.50	2.13	1.74	-18.51
Alaska	3.64	2.37	-34.77	92.85	64.93	-30.07	2.49	1.91	-23.21
Arizona	2.89	2.13	-26.41	78.62	58.44	-25.67	2.16	1.78	-17.30
Arkansas	3.36	2.36	-29.66	83.70	64.67	-22.73	2.25	1.94	-13.79
California	2.83	1.86	-34.46	72.24	50.66	-29.87	1.94	1.48	-23.59
Colorado	2.93	2.19	-25.24	79.40	60.38	-23.95	2.17	1.86	-14.08
Connecticut	2.53	1.79	-29.15	68.74	49.16	-28.49	1.83	1.46	-20.44
Delaware	2.72	1.86	-31.59	70.70	51.24	-27.52	1.89	1.51	-20.34
District of Columbia	3.94	1.94	-50.70	70.16	52.19	-25.61	1.92	1.63	-15.23
Florida	2.80	2.00	-28.76	76.09	55.31	-27.30	2.04	1.65	-19.24
Georgia	3.23	1.96	-39.29	75.81	53.99	-28.78	2.04	1.60	-21.60
Hawaii	2.55	1.91	-25.00	68.97	52.27	-24.22	1.85	1.53	-17.16
Idaho	2.94	2.09	-28.89	79.83	57.34	-28.17	2.13	1.71	-19.57
Illinois	2.85	1.84	-35.25	73.78	50.56	-31.46	1.99	1.49	-25.34
Indiana	2.65	1.93	-27.03	72.03	53.26	-26.07	1.93	1.57	-18.45
Iowa	2.65	2.05	-22.43	71.67	56.30	-21.45	1.93	1.70	-11.81
Kansas	2.77	2.09	-24.74	75.12	57.03	-24.07	2.02	1.72	-14.95
Kentucky	2.87	2.13	-25.74	77.96	58.89	-24.47	2.10	1.76	-16.19
Louisiana	3.12	2.13	-31.75	76.93	58.57	-23.87	2.07	1.69	-17.96
Maine	2.60	1.99	-23.27	70.67	54.67	-22.64	1.89	1.64	-13.55
Maryland	2.80	2.01	-28.06	72.04	55.23	-23.33	1.93	1.63	-15.50
Massachusetts	2.58	1.97	-23.92	70.25	53.82	-23.39	1.89	1.59	-15.87
Michigan	2.63	1.84	-30.04	67.82	50.81	-25.09	1.81	1.50	-17.07
Minnesota	2.79	2.03	-27.42	75.53	55.61	-26.37	2.04	1.71	-16.44
Mississippi	3.45	2.32	-32.78	79.72	60.50	-24.11	2.14	1.79	-16.22
Missouri	2.92	2.12	-27.41	78.98	58.19	-26.33	2.13	1.78	-16.46
Montana	3.07	2.38	-22.41	83.14	65.43	-21.30	2.18	1.92	-11.98
Nebraska	2.78	2.09	-24.64	75.23	57.54	-23.51	2.03	1.72	-15.42
Nevada	3.22	2.14	-33.56	82.89	58.37	-29.58	2.24	1.69	-24.33
New Hampshire	2.60	1.97	-24.21	70.74	54.36	-23.15	1.90	1.62	-14.56
New Jersey	2.39	1.74	-27.34	64.70	47.66	-26.34	1.73	1.39	-19.69
New Mexico	3.42	2.30	-32.91	84.63	63.04	-25.51	2.30	1.95	-15.18
New York	2.63	1.70	-35.47	66.63	46.68	-29.94	1.79	1.36	-24.26
North Carolina	2.93	1.93	-33.95	73.42	53.12	-27.64	1.99	1.64	-17.95
North Dakota	2.67	2.21	-17.20	72.14	60.57	-16.04	1.93	1.77	-8.15
Ohio	2.62	1.90	-27.42	70.89	52.47	-25.99	1.91	1.59	-17.06
Oklahoma	2.92	2.24	-23.29	78.58	61.76	-21.40	2.17	1.86	-14.14
Oregon	2.93	2.02	-31.10	79.66	55.60	-30.20	2.15	1.69	-21.13
Pennsylvania	2.57	1.88	-26.74	69.50	51.77	-25.51	1.86	1.55	-16.74
Rhode Island	2.56	1.84	-28.08	69.20	50.76	-26.64	1.87	1.52	-18.41
South Carolina	3.25	2.03	-37.60	74.85	55.79	-25.46	2.01	1.65	-17.68
South Dakota	2.90	2.39	-17.69	78.72	65.45	-16.86	2.11	1.92	-8.75
Tennessee	3.07	2.05	-33.34	77.31	56.51	-26.91	2.07	1.71	-17.45
Texas	3.01	1.99	-33.95	79.67	54.46	-31.64	2.15	1.62	-24.62
Utah	2.68	1.97	-26.58	73.04	54.09	-25.94	1.97	1.63	-17.18
Vermont	2.79	2.07	-25.57	75.27	56.95	-24.33	2.05	1.75	-14.63
Virginia	2.65	1.91	-27.93	71.87	52.32	-27.19	1.95	1.56	-20.16
Washington	2.76	1.89	-31.81	74.99	51.86	-30.84	2.02	1.59	-21.65
West Virginia	2.89	2.07	-28.31	74.01	57.32	-22.55	1.98	1.74	-12.30
Wisconsin	2.76	2.14	-22.61	74.76	58.66	-21.54	2.02	1.81	-10.53
Wyoming	3.05	2.32	-24.08	82.60	63.37	-23.28	2.20	1.86	-15.64

The five states with the lowest percent change in YLDs for unilateral UEA between 1990 and 2019 are North Dakota (-17.20%), South Dakota (-17.69%), Montana (-22.41%), Iowa (-22.43%), and Wisconsin (-22.61%). The five states with the lowest percent change in YLDs for bilateral UEA between 1990 and 2019 are North Dakota (-16.17%), South Dakota (-16.32%), Montana (-20.37%), Iowa (-20.93%), and Wisconsin (-21.27%). The five states with the highest percent change in YLDs for unilateral UEA between 1990 and 2019 are the District of Columbia (-50.70%), Georgia (-39.29%), South Carolina (-37.60%), New York (-35.47%), and Illinois (-35.25%). The five states with the highest percent change in YLDs for bilateral UEA between 1990 and 2019 are the District of Columbia (-61.60%), Georgia (-45.16%), South Carolina (-44.17%), Alabama (-39.56%), and New York (-38.23%). The five states with the lowest percent change in prevalence for unilateral UEA between 1990 and 2019 are North Dakota (-16.04%), South Dakota (-16.86%), Montana (-21.30%), Oklahoma (-21.40%), and Iowa (-21.45%). The five states with the lowest percent change in prevalence for bilateral UEA between 1990 and 2019 are North Dakota (-14.98%), South Dakota (-16.86%), Oklahoma (-21.40%), Montana (-21.30%), and Iowa (-20.13%). The five states with the highest percent change in prevalence for unilateral UEA between 1990 and 2019 are Texas (-31.64%), Illinois (-31.46%), Washington (-30.84%), Oregon (-30.20%), and Alaska (-30.07%). The five states with the highest percent change in prevalence for bilateral UEA between 1990 and 2019 are Illinois (-31.07%), Texas (-30.81%), Washington (-29.35%), New York (-29.04%), and California (-29.04%). The five states with the lowest percent change in incidence for unilateral UEA between 1990 and 2019 are North Dakota (-8.15%), South Dakota (-8.75%), Wisconsin (-10.53%), Iowa (-10.56%), Montana (-11.98%), and West Virginia (-12.30%). The five states with the lowest percent change in incidence for bilateral UEA between 1990 and 2019 are North Dakota (-6.91%), South Dakota (-7.24%), Wisconsin (-9.28%), Iowa (-11.81%), Montana (-10.67%), and West Virginia (-11.13%). The five states with the highest percent change in incidence for unilateral UEA between 1990 and 2019 are Illinois (-25.34%), Texas (-24.62%), Nevada (-24.33%), New York (-24.26%), and California (-23.59%). The five states with the highest percent change in incidence for bilateral UEA between 1990 and 2019 are Illinois (-24.84%), Texas (-24.15%), New York (23.41%), Nevada (-22.72%), and California (-22.51%).

## Discussion

The analysis showed that men consistently had higher rates of YLDs, incidence, and prevalence of both bilateral and unilateral UEAs compared to women from 1990 to 2019, across all regions of the US. This finding aligns with existing literature, which also indicates a higher ratio of men undergoing UEAs compared to women [7]. The primary causes of UEAs in adults (traumatic and occupational injuries) are more prevalent among men, likely due to their higher risk profiles, which may account for the increased YLDs, incidence, and prevalence in this demographic nationwide [1].

The Northeast has persistently higher UEA rates, potentially influenced by factors such as a historical industrial presence and urban density. This general pattern suggests occupational and environmental hazards may play a role in UEA prevalence. In contrast, the lower UEA rates in the South may relate to its economy, which relies on agriculture, where injury mechanisms are generally distinct from those in more industrialized regions. Non-industrial injuries, common in agricultural settings, may be contributing to the South’s lower amputation rates compared to regions with high-density manufacturing and construction industries [15,16]. However, much more importantly, there is also the likely underreporting of injuries at the lower end of the UEA scale due to limited healthcare access.

The Midwest has its combination of industrial and agricultural economies. States such as Ohio and Michigan have sizable automotive and manufacturing sectors, as well as automobile assembly plants. Workers there use machinery and plastics during production in a high-incident environment, thus elevating the UEA risk. The Midwest also has significant farm regions where high UEA risks come from agricultural injuries; all of these factors lead to a moderate overall UEA rate [4,17-21]. Over time, the Midwestern economy has diversified, but both the current risk factors and the region’s historical high-risk industrial exposure continue to lead to elevated UEA rates.

In the West, where logging, mining, and other traditional industries, as well as rapid urbanization, creating new industries and a culture of building, have contributed to UEA-related deaths, we see concentrations in places such as California and Nevada. The presence of immigrant workers, who make up a significant portion of the labor force in these states, working in agriculture and construction, and enduring hazardous conditions and limited access to healthcare, also contributes to making this occupational landscape a dangerous one, perpetuating high rates of UEA over time [22-25]. Based on our findings, there are distinctive regional patterns of UEA in the US, with conditions that strongly influence these rates being linked to those area-specific industrial, occupational, and socioeconomic factors. These relationships are important to recognize because they can help public health officials anticipate the occurrence of UEAs and develop evidence-based strategies for reducing incidences and improving outcomes for affected populations [26-28].

Overall, a comprehensive look at the data reveals a reduction in the burden of UEA in the US, with considerable decreases in YLDs, prevalence, and incidence over the last 30 years. Following trauma-related injuries, malignancies, and vascular complications are other significant causes of UEAs [29]. Additionally, being male is an inherent risk factor for developing peripheral vascular disease, and diabetes mellitus is more commonly found in men [16,30]. These factors, in combination, help contribute to higher rates of UEAs in men. This is especially true given the poor healing potential in both conditions and the increased risk of infections, including necrotizing infections, with diabetes and chronic open wounds. Every region and state has experienced this improvement, although some areas have fared better than others. The gender divide has also reduced, although men still have a higher burden of UEAs than women. These trends reflect successful public health interventions, improved preventive care, and better healthcare access throughout the country.

Clinical implications

Overall, the findings of this study hold several important implications for clinical practice. Recognizing that men and individuals in industrialized or high-risk occupational settings have disproportionately higher rates of UEAs allows clinicians to prioritize screening and preventive interventions in these groups. Awareness of regional trends can help healthcare providers tailor community-based injury prevention initiatives and enhance coordination with occupational health programs. Furthermore, understanding that vascular disease and diabetes remain key contributors to amputation risk emphasizes the importance of aggressive management of these conditions at the primary care level. Finally, by identifying the populations most affected, clinicians can advocate for improved access to prosthetic and rehabilitative services, psychosocial support, and patient education programs designed to optimize functional recovery and quality of life after amputation.

Limitations

This study has several limitations that must be acknowledged. As a retrospective analysis, there are inherent challenges related to the use of historical data. The medical records utilized were not originally designed for research, which can result in incomplete documentation or gaps in patient histories, potentially introducing bias and affecting the accuracy of the findings. Additionally, the GBD database is dependent on the medical coding and comprehensiveness of the data provided by healthcare facilities. Inconsistencies in coding and the possibility of omitting certain patient records can lead to variations and the potential under- or overreporting of UEA cases across the nation.

## Conclusions

This study provides a delineated temporal analysis of both YLDs and prevalence and incidence of UEAs over time in the United States between 1990 and 2019. The analysis revealed an overall decreasing trend in YLDs, prevalence, and incidence of UEAs across the country. Despite these beneficial outcomes, disparities persist, as men have higher rates of UEAs than women, and there are regional differences reflective of industrial, occupational, and health environment disparities. The Northeast had the highest overall UEA rates, possibly due to its earlier industrial base, while the South had the lowest rate, possibly due to its rural agricultural economy and historical secondary health environment. These findings reveal the need for public health programs led by epidemiological experts, policymakers, and especially orthopedic surgeons themselves to further reduce the burden of UEAs in the US and spread awareness of traffic safety, best practices in occupational safety, and general injury prevention with building codes, handrails, and other accessibility infrastructure. Finally, these initiatives should also focus on the remaining gender and regional gaps and include enhancing preventative efforts while improving access to care in regions with historically higher UEA rates.

## References

[1] Souza K, Cantley LF, Slade MD, Eisen EA, Christiani D, Cullen MR (2014). Individual-level and plant-level predictors of acute, traumatic occupational injuries in a manufacturing cohort. Occup Environ Med.

[2] McDonald CL, Westcott-McCoy S, Weaver MR, Haagsma J, Kartin D (2021). Global prevalence of traumatic non-fatal limb amputation. Prosthet Orthot Int.

[3] Solarz MK, Thoder JJ, Rehman S (2016). Management of major traumatic upper extremity amputations. Orthop Clin North Am.

[4] Warner M, Baker SP, Li G, Smith GS (1998). Acute traumatic injuries in automotive manufacturing. Am J Ind Med.

[5] Barancik JI, Chatterjee BF, Greene YC, Michenzi EM, Fife D (1983). Northeastern Ohio trauma study: I. Magnitude of the problem. Am J Public Health.

[6] Tintle SM, Baechler MF, Nanos GP 3rd, Forsberg JA, Potter BK (2010). Traumatic and trauma-related amputations: part II: upper extremity and future directions. J Bone Joint Surg Am.

[7] Inkellis E, Low EE, Langhammer C, Morshed S (2018). Incidence and characterization of major upper-extremity amputations in the National Trauma Data Bank. JB JS Open Access.

[8] Global Burden of Disease Collaborative Network. Global Burden of Disease Study 2021 (GBD (2024). Global Burden of Disease Collaborative Network: Global Burden of Disease 2021: findings from the GBD 2021 Study. https://www.healthdata.org/research-analysis/library/global-burden-disease-2021-findings-gbd-2021-study.

[9] Coon M, Denisiuk M, Woodbury D, Best B, Vaidya R (2022). Closed fracture treatment in adults, when is it still relevant?. Spartan Med Res J.

[10] Amin S, Achenbach SJ, Atkinson EJ, Khosla S, Melton LJ 3rd (2014). Trends in fracture incidence: a population-based study over 20 years. J Bone Miner Res.

[11] Singaram S, Naidoo M (2019). The physical, psychological and social impact of long bone fractures on adults: a review. Afr J Prim Health Care Fam Med.

[12] Farr JN, Melton LJ 3rd, Achenbach SJ, Atkinson EJ, Khosla S, Amin S (2017). Fracture incidence and characteristics in young adults aged 18 to 49 years: a population-based study. J Bone Miner Res.

[13] (2024). U.S. Census Bureau: Census regions and divisions of the United States. https://www2.census.gov/geo/pdfs/maps-data/maps/reference/us_regdiv.pdf.

[14] Rabah NM, Knusel KD, Khan HA, Marcus RE (2020). Are there nationwide socioeconomic and demographic disparities in the use of outpatient orthopaedic services?. Clin Orthop Relat Res.

[15] Wu VS, Schmidt JE, Jella TK, Cwalina TB, Freidl SL, Pumo TJ, Kamath AF (2023). Rural communities in the United States face persistent disparities in access to orthopaedic surgical care. Iowa Orthop J.

[16] Kautzky-Willer A, Harreiter J, Pacini G (2016). Sex and gender differences in risk, pathophysiology and complications of type 2 diabetes mellitus. Endocr Rev.

[17] Largo TW, Rosenman KD (2015). Surveillance of work-related amputations in Michigan using multiple data sources: results for 2006-2012. Occup Environ Med.

[18] Schweizer MA, Janak JC, Graham B (2019). Nonfatal motor vehicle related injuries among deployed US Service members: characteristics, trends, and risks for limb amputations. J Trauma Acute Care Surg.

[19] Reilly MJ, Wang L, Rosenman KD (2024). Evaluation of the characteristics of injured workers and employer compliance with OSHA's reporting requirement for work-related amputations. Am J Ind Med.

[20] (2024). Katz, Friedman, Eisenstein, Johnson, Bareck & Bertuca: Amputation injuries in the auto industry. https://www.kfeej.com/practice-areas/workers-compensation/auto-industry-workers-compensation-claims/amputation-injuries-in-the-auto-industry/.

[21] Schneble CA, Raymond J, Loder RT (2019). The demographics of non-motor vehicle associated railway injuries seen at trauma centers in the United States 2007 - 2014. Cureus.

[22] Shankar J, Lai D, Chen SP, Turin TC, Joseph S, Mi E (2022). Highly educated immigrant workers’ perspectives of occupational health and safety and work conditions that challenge work safety. Int J Environ Res Public Health.

[23] Porru S, Baldo M (2022). Occupational health and safety and migrant workers: has something changed in the last few years?. Int J Environ Res Public Health.

[24] Inglesby DC, Okewunmi J, Williams CS, Gopman JM, Melamed E (2022). Hand and upper extremity trauma in the undocumented immigrant population in the United States. Plast Reconstr Surg Glob Open.

[25] (1995). Comparing the incidence of lower extremity amputations across the world: the Global Lower Extremity Amputation Study. Diabet Med.

[26] Molina CS, Faulk J (2025). Lower extremity amputation. StatPearls.

[27] Chen K, Duan GY, Wolf JM, Stepan JG (2023). Health disparities in hand and upper extremity surgery: a scoping review. J Hand Surg Am.

[28] Orr AE (2020). Rehabilitation for persons with upper extremity amputation. Orthotics and Prosthetics in Rehabilitation.

[29] Maduri P, Akhondi H (2025). Upper limb amputation. StatPearls.

[30] Swenty CF, Hall M (2020). Peripheral vascular disease. Home Healthc Now.

